# Konservative Kombinationstherapie beim lumbalen Bandscheibenvorfall mit Nervenwurzelreizsyndrom mit mechanischer Physiotherapie (McKenzie), Gabapentin und transforaminalen epiduralen Infiltrationen

**DOI:** 10.1007/s00482-024-00824-y

**Published:** 2024-08-12

**Authors:** Manuela Jäntsch-Rieckert, Oliver Rommel, Verena Kästner, Lotte Maercklin-Rommel, Georg Jäger

**Affiliations:** 1 Abteilung für Neurologie und Schmerztherapie, Rommel-Klinik GmbH, Bätznerstraße 96–98, 75323 Bad Wildbad, Deutschland; 2 Abteilung für Orthopädie und Schmerztherapie, Rommel-Klinik GmbH, Bad Wildbad, Deutschland

**Keywords:** Nucleous pulposus, Wurzelaffektion, Antikonvulsiva, Infiltrationen, Krankengymnastik, Nucleous pulposus, Root affection, Anticonvulsants, Infiltrations, Physiotherapy

## Abstract

**Hintergrund:**

Die Behandlung des Nervenwurzelreizsyndroms beim lumbalen Bandscheibenvorfall kann chirurgisch oder konservativ erfolgen. Die konservativen Behandlungskonzepte zeigten einen Erfolg bei 90 % der Patienten, in der Mehrzahl der Studien wurde jedoch kein strukturiertes Behandlungskonzept angewendet.

**Ziel der Arbeit:**

In der Studie untersuchten wir den Effekt einer kombinierten konservativen Therapie des Wurzelreizsyndroms mit mechanischer Physiotherapie (McKenzie), Gabapentin sowie transforaminalen epiduralen Kortikosteroidinjektionen bei 40 Patienten während eines 10-tägigen stationären Aufenthalts.

**Methoden:**

Neben der klinischen Untersuchung einschließlich des Straight-leg-raise-Tests und Finger-Boden-Abstands wurde die Schmerzstärke in Ruhe und nach einer Gehstrecke von 5 m erhoben und eine Elektromyographie der Kennmuskeln durchgeführt. Die Oswestry Pain Disability Scale sowie Schmerzschwereskala nach von Korff wurden erhoben und neuropathische Schmerzkomponenten mit der painDETECT-Skala erfasst. Ferner wurden die Dauer der Arbeitsunfähigkeit sowie die Notwendigkeit einer Operation innerhalb des Beobachtungszeitraums erfasst. Die Untersuchungen wurden am Tag der Aufnahme, an den Tagen 3, 6 und 10 sowie 3 Monate nach Entlassung durchgeführt.

**Ergebnisse:**

Während der Behandlung konnte eine kontinuierliche Schmerzreduktion in Ruhe sowie beim Gehen von 5 m, Verbesserung des Straight-leg-raise-Tests sowie Verminderung des Finger-Boden-Abstands dokumentiert werden. Da die 3 Verfahren mit zeitlichem Abstand eingesetzt wurden, konnte gezeigt werden, dass alle signifikant zur Besserung beitrugen. Alle 3 Verfahren wurden ohne wesentliche Nebenwirkungen vertragen und die anhaltende Besserung bei der Nachuntersuchung nach 12 Wochen bestätigte die Nachhaltigkeit des Konzepts. Bei Aufnahme zeigten 32 % der Patienten eine überwiegend neuropathische Schmerzkomponente, welche nach 3 Monaten auf 7 % reduziert war. In der Elektromyographie fanden sich bei Aufnahme bei 70 % der Patienten Auffälligkeiten. Eine Kraftminderung zeigte sich bei Aufnahme bei 28 Patienten, bei der Kontrolle nur noch bei 7 Patienten. Auch die Lebensqualität war signifikant verbessert und die Patienten konnten 5,8 Wochen nach Behandlungsbeginn die Berufstätigkeit wieder aufnehmen. 3 von 40 Patienten benötigten wegen anhaltender Schmerzen eine Operation.

**Konsequenz:**

Das untersuchte kombinierte Behandlungsprogramm ist effektiv und wird gut toleriert.

## Abstract (en)

**Background:**

Treatment of sciatica due to lumbar disc herniation can be surgical or conservative. Conservative management has been described to be effective in 90% of patients; however, in most studies no consistent treatment concept was used.

**Objectives:**

In the present study, we evaluated the effect of a combined nonsurgical management (McKenzie physiotherapy, gabapentin, and periradicular injections) in 40 patients during a 10-day inpatient treatment.

**Methods:**

In addition to the neuro-orthopedic examination, pain severity at rest and after walking were assessed. The Oswestry pain disability scale, the pain severity scale, and the painDETECT scale were examined to assess neuropathic pain components. The duration of incapacity for work and the requirement of a later surgery were recorded. Examinations were performed on the day of admission, on days 3, 6, 10, and 84, 3 months after discharge.

**Results:**

During conservative treatment, a continuous reduction of pain and an improvement of the straight leg raise test as well as finger-to-floor distance could be documented. As the three treatment options were introduced with a time delay, it could be demonstrated that all significantly contributed to the improvement. All treatments were tolerated without side-effects and persistent improvement after 12 weeks. On admission, 32% of patients revealed a neuropathic pain component which decreased to 7% at the follow-up. A total of 28 patients showed impaired muscle strength on admission, which decreased to 7 patients on follow-up. Electromyography revealed pathological results in 70% of patients examined. A significant improvement of quality of life (Oswestry Disability Index [ODI]) could be observed and the patients returned to work after 5.8 weeks. Only 3/40 patients required surgical management due to persistent pain.

**Conclusion:**

The combined nonsurgical operative treatment program is effective and well tolerated.

## Hintergrund und Fragestellung

Der lumbale Bandscheibenvorfall kann durch Druck auf Spinalnerven zu einem radikulären Kompressionssyndrom mit Sensibilitätsstörung, Kraftminderung und Reflexabschwächung führen. Die Behandlung kann konservativ oder operativ sein. Die Nukleotomie ist einer der häufigsten chirurgischen Eingriffe bei Patienten mit Lumboischialgie in den USA und in Europa. Allerdings kann ein Bandscheibenvorfall auch bei Gesunden auftreten und sich ohne chirurgischen Eingriff zurückbilden [16]. *Die konservative Behandlung wurde in Übersichtsarbeiten bei über 80* *% als effektiv beschrieben *[18]*, mit physikalischer Therapie ließ sich bei 90* *% der Patienten ein gutes Behandlungsergebnis mit 92* *% Rückkehrquote zur Arbeit ermitteln *[28]*. Ältere Studien, welche konservative und operative Behandlung verglichen, berichteten über einen signifikant besseren Beschwerderückgang bei operierten Patienten beim Follow-up nach einem Jahr, wobei die Unterschiede nach 5 Jahren rückläufig waren *[1, 2]*.* Studien der letzten Jahre zeigten eine signifikante Verbesserung der Symptome bei operativ sowie konservativ behandelten Patienten, wobei sich durch die Operation eine schnellere Schmerzreduktion erzielen ließ. Nach einem Jahr zeigte sich jedoch kein signifikanter Unterschied zwischen den beiden Behandlungsgruppen, auch die Zahl ungünstiger Verläufe war nicht unterschiedlich [15, 24, 25, 32]. *In einer neuen Metaanalyse wurde eine schnellere Beschwerdelinderung im Zeitraum bis zu einem Jahr bestätigt, bei Follow-up-Untersuchungen nach 1 bis 8 Jahren fanden sich keine Unterschiede zwischen konservativer und operativer Behandlung *[17]*. *Allerdings wurden in allen genannten Studien keine strukturierten konservativen Behandlungskonzepte eingesetzt. Manche Patienten bekamen Infiltrationen, andere Medikamente und die physiotherapeutischen Techniken wurden nicht genauer beschrieben. Dies resultierte auch aus der geringen Evidenz der Wirksamkeit konservativer Behandlungen. Allerdings wurden in den letzten 20 Jahren mehrere Studien publiziert, welche eine Verbesserung lumbaler Schmerzen nach Behandlung mit der McKenzie-Methode zeigten [6, 20, 23]. Bei dieser Behandlungsmethode werden nach ausführlicher Untersuchung durch einen zertifizierten Therapeuten Bewegungen herausgearbeitet, bei welchen es zu einem Rückgang von Rückenschmerzen und Schmerzausstrahlung („Zentralisierung“) kommt und welche anschließend alle 2 h je 10-mal wiederholt werden müssen. Speziell für radikuläre Schmerzen wurde mechanische Physiotherapie in Anlehnung an das McKenzie-Konzept eingesetzt und führte zu einem stabilen Rückgang radikulärer Schmerzen [4, 5]. Gabapentin als Antikonvulsivum mit Wirksamkeit bei neuropathischen Schmerzen zeigte in einigen Studien einen guten Effekt bei Wurzelreizsyndromen [13, 22, 33]. Ferner wurde in mehreren Studien ein Schmerzrückgang nach bildwandlergestützten Kortikosteroidinfiltrationen beschrieben [12, 14, 29]. Daher wurden Patienten mit Bandscheibenvorfall und Radikulopathie über die letzten 10 Jahre in unserem neuroorthopädischen Akutkrankenhaus regelhaft mit einer Kombination aus Gabapentin, Physiotherapie nach McKenzie und bildwandlerkontrollierten transforaminalen epiduralen Infiltrationen behandelt. Das Ziel der vorliegenden Studie war es, die Wirksamkeit und Verträglichkeit dieser Behandlungskombination zu untersuchen.

## Studiendesign und Untersuchungsmethoden

### Rekrutierung der Patienten

Es handelt sich um eine prospektive Beobachtungsstudie, welche auf dem routinemäßigen Vorgehen der Abteilungen für Neurologie und Orthopädie der Rommel-Klinik Bad Wildbad basiert. Das Studienprotokoll wurde von der Ethikkommission der Landesärztekammer Baden-Württemberg (AZ-2016-075) genehmigt. 40 Patienten (männlich/weiblich 19/21, Alter 49 ± 13 Jahre) wurden in die Studie aufgenommen. Die Untersuchungen fanden zwischen November 2016 und Juni 2019 statt. Eingeschlossen wurden Patienten *im Alter von 18 bis 80 Jahren* mit lumbalem Bandscheibenvorfall und Wurzelreizung, welche zwischen 3 Wochen und 5 Monaten vor Aufnahme bestand. Die Diagnose wurde mit MRT oder CT bestätigt, welche einen Bandscheibenvorfall zeigte, der zu den klinischen Symptomen der Patienten passte. In die Studie wurden alle Patienten eingeschlossen, die mit einem standardisierten Behandlungsprogramm sowie Verlaufskontrollen am Tag der Aufnahme, an Tag 3, 6 und 10 sowie nach 3 Monaten (Tag 84) einverstanden waren.

### Klinische und technische Untersuchungen sowie Fragebögen

Bei der Aufnahme erfolgte bei allen Patienten eine klinische Untersuchung der Muskelkraft (0–5/5) sowie Sensibilität in Dermatomverteilung. Der Finger-Boden-Abstand (Zentimeter) und der Straight-leg-raise-Test (Winkelgrade) wurden gemessen. Alle Patienten bekamen eine Elektromyographie der Kennmuskeln L3 bis S1. Um die funktionelle Beeinträchtigung zu erfassen, wurde der Oswestry Disability Questionnaire (ODI), ferner die Schmerzschwereskala [8] angewendet. Um neuropathische Schmerzkomponenten zu erfassen, wurde der painDETECT-Score [9] eingesetzt. Zur Erfassung einer überlagernden Depression wurde die allgemeine Depressionsskala (ADS) angewendet. Die Schmerzstärke wurde auf einer numerischen Rating-Skala [1–10] in Ruhe und nach dem Gehen einer Strecke von 5 m erfasst. Alle klinischen Parameter sowie der ODI wurden bei Aufnahme, an Tag 6 und Tag 10 vor Entlassung erfasst. Die painDETECT-Skala sowie die allgemeine Depressionsskala wurden bei Aufnahme und nach 12 Wochen erhoben. Bei der Kontrolle nach 12 Wochen wurden die Schmerzstärke nach dem Gehen einer Strecke von 5 m, die Dauer der Arbeitsunfähigkeit sowie die Notwendigkeit einer Operation erfragt und eine klinische Untersuchung durchgeführt.

### Therapie

Am Tag der Aufnahme wurden alle Patienten von einem zertifizierten McKenzie-Therapeuten über eine Stunde untersucht. Nach den Ergebnissen erhielt jeder Patient Übungen, welche alle 2 h 10 × wiederholt werden mussten. Dies wurde über 3 Tage weitergeführt, am 3. Tag wurde die Untersuchung durch den McKenzie-Therapeuten wiederholt und der Effekt hinsichtlich Schmerzintensität in Ruhe, SLR sowie Muskelkraft erfasst. Die Übungen wurden während des gesamten Aufenthalts weitergeführt.

Bei Aufnahme wurde die bisherige Schmerzmedikation beendet und auf Novaminsulfon 500 mg 3 × täglich umgestellt, welches mit der Aufdosierung von Gabapentin und dem Schmerzrückgang zum Ende des stationären Aufenthalts wieder abgesetzt wurde. Am Abend des 3. Tags wurde Gabapentin mit 300 mg begonnen und an den Folgetagen in 300 mg Schritten/Tag erhöht, sodass die Zieldosis von 3 × 600 mg am 8. Tag erreicht war.

*Am 7. und 9. Tag wurden bildwandlergestützt fluoroskopische transforaminale epidurale Injektionen mit 5* *ml Bupivacain 0,25* *% sowie Triamcinolon 40* *mg durchgeführt.*

### Statistik

#### Die Statistik erfolgte mithilfe des Open-Source-Programms „R“ in der Version 3.6.1.

Um die Signifikanz der Unterschiede zwischen an verschiedenen Tagen erhobenen Messwerteverteilungen zu beurteilen, wurde für jeden Parameter ein Friedman-Test durchgeführt. Die im Verlauf beobachteten Verteilungsverschiebungen wurden nur als statistisch signifikant eingestuft, wenn aus dem Test ein *p*-Wert < 0,05 resultierte.

## Ergebnisse

### Verträglichkeit der Therapiemaßnahmen

Die mechanische Physiotherapie nach McKenzie wurde gut vertragen**. ***5 Patienten beklagten eine Zunahme der Schmerzen nach den ersten Übungen, welche zu einer Anpassung der Übungen durch den McKenzie-Therapeuten mit Reduktion der Intensität, Verlängerung der Intervalle oder der Anzahl der Wiederholungen führte, jedoch in keinem Fall zum Abbruch der Therapie.* Gabapentin konnte bei allen Patienten auf 1800 mg/Tag aufdosiert werden. Bei 36 von 40 Patienten wurde es ohne Nebenwirkungen toleriert. Bei 4 Patienten zeigten sich in den ersten Tagen geringe Nebenwirkungen (Schwindel *N* = 2, Kopfschmerz und Übelkeit *N* = 1, Gedächtnisstörungen und Unruhe *N* = 1), welche sich nach einigen Tagen besserten, sodass alle Patienten mit der Zieldosis über 70 Tage behandelt werden konnten. Die transforaminalen epiduralen Infiltrationen wurden von allen Patienten gut toleriert. Während der Infiltration beklagten viele Patienten einen „memory pain“, welcher in das Bein ausstrahlte, und eine Taubheit für einige Stunden. Der radikuläre Schmerz bildete sich nach der Injektion zurück, sodass alle Patienten mit einer 2. Infiltration einverstanden waren.

### Schmerz in Ruhe und nach dem Gehen einer Strecke von 5 m

**Abb. 1:**
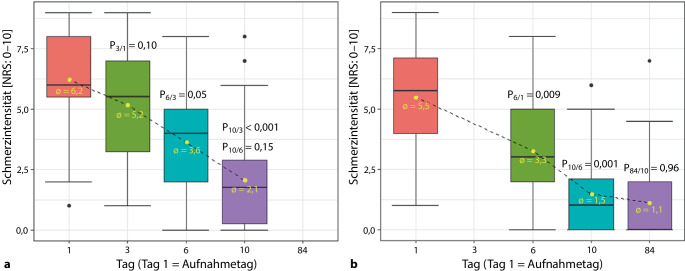
Schmerzstärke (NRS 0–10) in Ruhe (**a**) und nach dem Gehen von 5 m (**b**) bei Aufnahme (Tag 1), nach McKenzie-Physiotherapie (Tag 3), nach kombinierter Behandlung McKenzie/Gabapentin (Tag 6), nach zusätzlicher transforaminaler epiduraler Infiltration (Tag 10) und bei der Verlaufskontrolle (Tag 84)

Während des stationären Aufenthalts zeigte sich eine signifikante Reduktion der Schmerzstärke in Ruhe (SR) sowie nach dem Gehen von 5 m (SG). Obwohl eine Ruheschmerzreduktion bereits nach Durchführung der Physiotherapie nach McKenzie (Tag 3) zu beobachten war, zeigte sich ein signifikanter Effekt erst nach zusätzlicher Behandlung mit Gabapentin (*p* ≤ 0,05). Nach zusätzlicher Infiltration war der Ruheschmerz nicht signifikant gebessert, der Schmerz beim Gehen verglichen zur McKenzie‑/Gabapentin-Behandlung jedoch signifikant vermindert (*p* = 0,001, Abb. 1a, b). Bei der Kontrolle nach 12 Wochen bestätigte sich beim Gehen von 5 m eine anhaltende Schmerzreduktion, was die Nachhaltigkeit der Behandlung untermauerte (Schmerzintensität bei Aufnahme 5,5/10, an Tag 84 1,1/10, Abb. 1b).

### Finger-Boden-Abstand

**Abb. 2:**
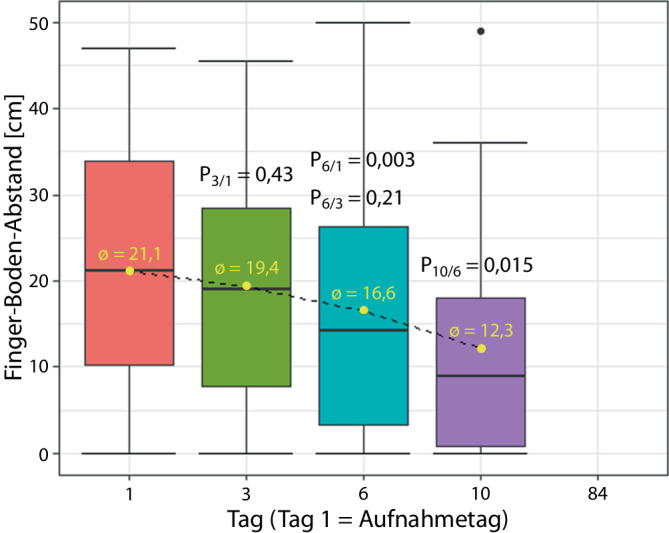
Finger-Boden-Abstand (in Zentimetern) bei Aufnahme (Tag 1), nach McKenzie-Physiotherapie (Tag 3), nach kombinierter Behandlung McKenzie/Gabapentin (Tag 6) und nach zusätzlicher transforaminaler epiduraler Infiltration (Tag 10)

Der Finger-Boden-Abstand (FBA) zeigte eine konstante Verbesserung zwischen Tag 1 und Tag 10, wobei das höchste Signifikanzlevel zwischen Tag 1 und Tag 6 (nach McKenzie und Gabapentin, *p* ≤ 0,003) zu erreichen war. Dennoch erbrachten auch die transforaminalen epiduralen Infiltrationen einen zusätzlich signifikanten Effekt (*p* ≤ 0,015, Abb. 2).

### Straight-leg-raise-Test (SLR)

**Abb. 3:**
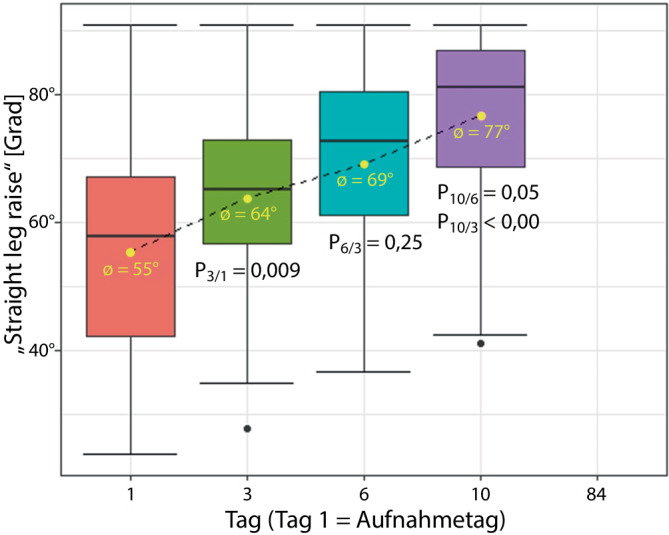
Lasègue-Manöver („straight leg raise“) (in Grad) bei Aufnahme (Tag 1), nach McKenzie-Physiotherapie (Tag 3), nach kombinierter Behandlung McKenzie/Gabapentin (Tag 6) und nach zusätzlicher transforaminaler epiduraler Infiltration (Tag 10)

Der SLR zeigte eine konstante Verbesserung zwischen Aufnahme und Entlassung, welche am auffälligsten zwischen Tag 1 und Tag 3 nach der Physiotherapie nach McKenzie war (*p* ≤ 0,009), wogegen die zusätzliche Behandlung mit Gabapentin keine signifikante Besserung erbrachte. Allerdings konnte nach den transforaminalen epiduralen Infiltrationen eine weitere signifikante Verbesserung beobachtet werden (*p* ≤ 0,05 zwischen Tag 10 und Tag 6, Abb. 3).

### Oswestry Disability Index Score

**Abb. 4:**
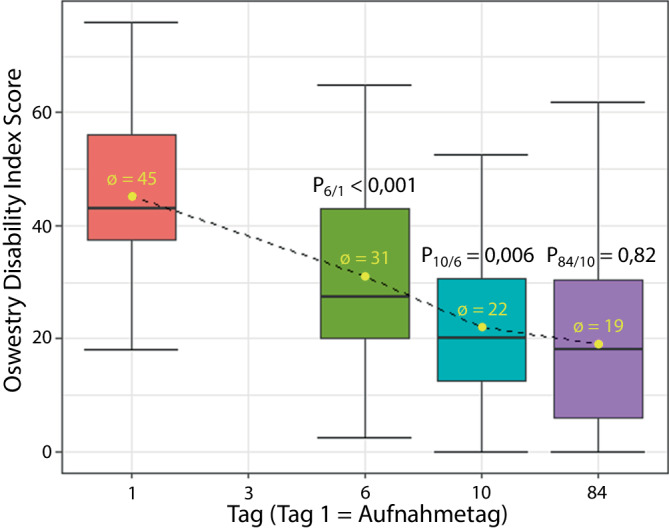
Der Oswestry Disability Index Score bei Aufnahme (Tag 1), nach kombinierter Behandlung McKenzie/Gabapentin (Tag 6), nach zusätzlicher transforaminaler epiduraler Infiltration (Tag 10) und bei der Verlaufskontrolle (Tag 84)

Die Lebensqualität konnte signifikant zwischen Tag 1 und Tag 6 (*p* ≤ 0,001) sowie Tag 6 und Tag 10 (*p* ≤ 0,006) verbessert werden, mit bleibender Besserung nach 12 Wochen (Abb. 4).

### Neuropathische Schmerzkomponente (painDETECT)

**Abb. 5:**
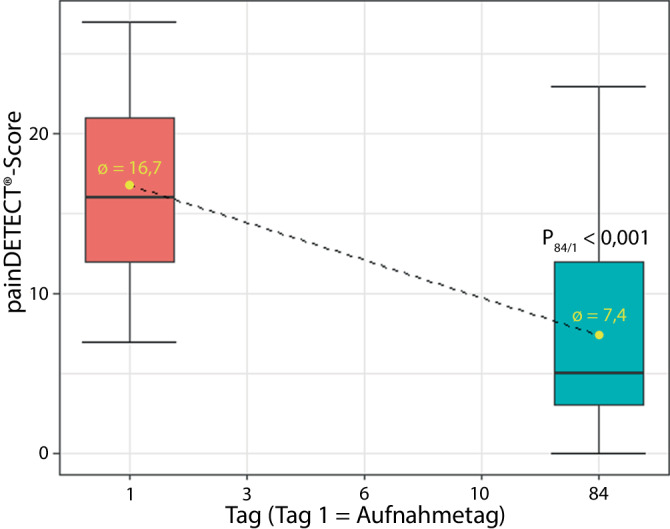
Der painDETECT-Score bei Aufnahme (Tag 1) und bei der Verlaufskontrolle (Tag 84)

Bei Aufnahme hatten 33 % der Patienten eine neuropathische Schmerzkomponente mit einem painDETECT-Score (0–38) von über 19. Der mittlere painDETECT-Score aller Patienten war 16,7 und war nach 12 Wochen signifikant auf 7,4 reduziert (*p* ≤ 0,001), und nur noch 8 % der Patienten erfüllten die Kriterien eines neuropathischen Schmerzes (Abb. 5).

### Kraft der Kennmuskeln

Bei Aufnahme hatten 28 von 40 Patienten (70 %) eine Kraftminderung der Kennmuskeln der betroffenen Nervenwurzel. Unter diesen Patienten hatten 11 Patienten eine Muskelkraft < 4,0/5 (4 Patienten < 3,0/5). Bei diesen Patienten wurde als Behandlungsalternative eine Operation vorgeschlagen, alle Patienten zogen es vor, die konservative Behandlung fortzusetzen. Nach 10 Behandlungstagen war die Kraftminderung bei 13 von 28 Patienten (46 %) vollkommen zurückgebildet. Bei 8 von 28 Patienten (29 %) bildete sich die Kraftminderung innerhalb von 12 Wochen zurück, wogegen 7 Patienten eine Kraftminderung behielten.

### Elektromyographie (EMG)

Die EMG der Kennmuskeln zeigte bei 12/40 Patienten Zeichen einer akuten Denervierung, bei 16/40 Patienten chronisch-neurogene Veränderungen und bei 4/40 Patienten sowohl akute wie auch chronische Auffälligkeiten. 10/12 Patienten mit akuter Denervierung hatten eine Kraftminderung der Kennmuskulatur, bei 2/12 Patienten fand sich keine motorische Beeinträchtigung. 5/10 Patienten mit Denervierung in der EMG und Kraftminderung hatten eine komplette Rückbildung der Kraftminderung bis zur Krankenhausentlassung.

### Sensibilitätsstörungen

37 von 40 Patienten (92 %) hatten eine radikuläre Sensibilitätsstörung in den betroffenen Dermatomen. Diese bildete sich bei 19/37 Patienten (51 %) bis zur Entlassung zurück, bei 3/37 Patienten (8 %) bis zur Kontrolle nach 12 Wochen, 15/37 Patienten hatten eine bleibende Sensibilitätsstörung.

### Komorbide depressive Störung

Bei 2 Patienten waren die Kriterien einer depressiven Episode erfüllt. Ein Patient hatte eine milde depressive Episode, welche sich im Lauf der Behandlung besserte, weshalb der Patient 2 Wochen nach Krankenhausentlassung wieder arbeiten konnte. Der 2. Patient zeigte Symptome einer schwereren Depression und war für 8 Wochen nach der Entlassung noch arbeitsunfähig. Bei beiden Patienten war keine Operation erforderlich.

### Schmerzschweregrad nach von Korff

33 von 40 Patienten (83 %) erfüllten am Aufnahmetag die Kriterien einer schweren schmerzassoziierten Beeinträchtigung (Grad 4 nach von Korff; [31]). Nach 12 Wochen hatten lediglich 6 von 40 Patienten noch ein höheres Level (Grad 3 und 4), wogegen die Mehrheit der Patienten (34/40) nur eine geringe oder keine schmerzassoziierte Beeinträchtigung (Grad 1, *N* = 30, Grad 0, *N* = 4) berichtete.

### Arbeitsunfähigkeit

Die durchschnittliche Dauer der Arbeitsunfähigkeit nach Krankenhausentlassung war 5,8 Wochen (± 4,4 Wochen). 9 der 40 Patienten benötigten eine stationäre Anschlussheilbehandlung.

### Poststationäre chirurgische Intervention

Bei 3 von 40 Teilnehmern der Studie war eine Operation innerhalb von 12 Wochen erforderlich. Bei allen Patienten war nicht die neurologische Beeinträchtigung, sondern der anhaltende Schmerz Grund für die Operation. Alle 3 Patienten hatten im Anschluss eine Besserung der Schmerzintensität und Beeinträchtigung und waren wieder arbeitsfähig.

## Diskussion

In der vorliegenden Studie untersuchten wir den Effekt eines konservativen Behandlungskonzepts aus Physiotherapie nach McKenzie, Gabapentin und transforaminalen epiduralen Kortikosteroidinjektionen bei 40 Patienten mit lumbalem Bandscheibenvorfall und Radikulopathie während eines 10-tägigen stationären Aufenthalts. Durch die Behandlung konnten eine kontinuierliche Reduktion des Ruheschmerzes und des Schmerzes beim Gehen von 5 m sowie eine Besserung im SLR sowie Finger-Boden-Abstand erreicht werden. Da die 3 Behandlungsverfahren mit zeitlichem Abstand hintereinander begonnen wurden, konnte gezeigt werden, dass alle signifikant zur Verbesserung beitrugen. Alle 3 Verfahren wurden gut vertragen und die bleibende Verbesserung bei der Kontrolle nach 12 Wochen bestätigte die Nachhaltigkeit des Konzepts.

Bei einem Bandscheibenvorfall besteht ein gemischtes Schmerzsyndrom aus nozizeptiven, neuropathischen und mechanischen Komponenten, wobei die mechanische Komponente durch die McKenzie-Physiotherapie, der neuropathische Schmerz durch Gabapentin sowie die nozizeptiv-inflammatorische Komponente über die Kortikosteroidinfiltration beeinflusst werden kann.

Während am Aufnahmetag 32 % der Patienten im painDETECT-Score Hinweise auf einen überwiegend neuropathischen Schmerz zeigten, fand sich dies bei der Kontrolle nach 3 Monaten nur noch bei 7 % der Patienten. Dies bestätigt die Bedeutung der neuropathischen Schmerzkomponente bei der lumbalen Radikulopathie. Viele Medikamente wie NSAR, Pregabalin, Opioide oder trizyklische Antidepressiva zeigten bei der lumbalen Radikulopathie keine bessere Wirksamkeit als Placebo [3, 10, 26, 27]. *Zum Einsatz von Gabapentin bei der Radikulopathie gibt es eine placebokontrollierte Studie, in welcher Gabapentin signifikant besser als Placebo radikuläre Schmerzen sowie motorische und sensible Defizite linderte *[33]. In einer weiteren Studie bei 78 Patienten mit akuter sowie chronischer Ischialgie zeigte sich über 3 Monate eine signifikante Verbesserung von Schmerzen und Funktion [13], wobei lediglich 6 von 78 Patienten Gabapentin wegen Nebenwirkungen beendeten. Unsere Ergebnisse bestätigen diese Beobachtungen.

Daneben zeigte sich in der vorliegenden Studie eine deutliche Besserung neurologischer Defizite: Während 28 Patienten bei Aufnahme eine Kraftminderung aufwiesen, ließ sich dies bei der Verlaufsuntersuchung nur noch bei 7 Patienten nachweisen. 70 % der Patienten zeigten bei der Aufnahme Auffälligkeiten in der Elektromyographie, davon 12 Patienten mit akuter Denervierung, wovon jedoch 2 keine Kraftminderung hatten. Bei diesen Patienten war die Elektromyographie hilfreich für die Entscheidung, welche Nervenwurzel für die transforaminale epidurale Infiltration gewählt wurde.

Im Hinblick auf die wissenschaftliche Datenlage muss hinterfragt werden, wann eine Operation beim Bandscheibenvorfall mit Kraftminderung erforderlich ist. Studien, welche konservative und operative Behandlungsoptionen verglichen, zeigten keine signifikanten Unterschiede bezüglich der Frequenz ungünstiger Verläufe. Obwohl bei frühem chirurgischem Vorgehen eine schnellere Erholung des motorischen Defizits beobachtet wurde, war der Unterschied nach einem Jahr nicht mehr signifikant [21]. Nach unserer Kenntnis verglich nur eine Studie die Rückbildung einer Kraftminderung nach Bandscheibenvorfall zwischen operativer und konservativer Behandlung [7]. Hier wurden 67 Patienten mit Bandscheibenvorfall und einer Parese ≤ 3/5 für weniger als einen Monat chirurgisch oder konservativ behandelt. Hinsichtlich der Paresen zeigte sich kein Unterschied zwischen den beiden Behandlungsoptionen und eine Verbesserung der motorischen Funktionen konnte in beiden Gruppen beobachtet werden. Insofern ist auch bei vorliegenden Kraftminderungen nach Aufklärung des Patienten ein konservativer Behandlungsversuch gerechtfertigt.

Mechanische Physiotherapie nach dem McKenzie-Konzept ist zuverlässig und eröffnet den Patienten die Möglichkeit, den Schmerz und den Behandlungsverlauf selbst zu beeinflussen, indem wiederholt Bewegungen durchgeführt werden, welche den Druck der Bandscheibe auf die Nervenwurzel reduzieren sollen [19]. Das hohe Maß an Selbstwirksamkeit stellt einen Gegensatz zu den häufig passiven physiotherapeutischen Behandlungsoptionen bei Rückenschmerzen dar. Der Aspekt, dass die meisten Patienten dankbar für eine aktivere Rolle in der Behandlung sind, könnte zu den günstigen Ergebnissen in dieser Studie beitragen. Wissenschaftliche Studien zur mechanischen Physiotherapie in Anlehnung an das McKenzie-Konzept (Tübinger Konzept) zeigten bei einem hohen Prozentsatz von Patienten eine Besserung der Symptome, obwohl sich bei Kernspinkontrollen keine Änderung der Größe des Bandscheibenvorfalls zeigte [5]. Innerhalb der Beobachtungszeit von 5 Jahren benötigten lediglich 6 von 50 Patienten, welche die mechanische Physiotherapie regelmäßig durchführten, eine Operation.

Bezüglich fluoroskopischer epiduraler oder transforaminaler epiduraler Injektionen von Kortikosteroiden zur Behandlung radikulärer Schmerzen zeigte sich in 2 *wissenschaftlichen Arbeiten* bei 75 % bzw. 64 % der Patienten eine Besserung der Schmerzintensität von mehr als 50 % [12, 29]. In einer dieser *Publikationen* fand sich eine anhaltende Verbesserung bei der Kontrolle nach einem Jahr [29], wogegen in der anderen *Publikation*, welche den kurzzeitig positiven Effekt bestätigte, nach 5 Jahren eine große Zahl von Rückfällen beschrieben wurde [14]. Nach der S2k-Leitlinie der Deutschen Gesellschaft für Orthopädie und Unfallchirurgie werden transforaminale epidurale Injektionen als Behandlung für die akute und subakute Radikulopathie beim Bandscheibenvorfall empfohlen [11].

Eine signifikante Besserung zeigte sich auch bezüglich der Lebensqualität und die Patienten konnten im Durchschnitt 5,8 Wochen nach der Krankenhausentlassung wieder zur Arbeit zurückkehren. Nur 3 Patienten mussten wegen persistierender Schmerzen operiert werden und erholten sich komplett nach der Operation.

Obwohl die deutschen Leitlinien für die Behandlung radikulärer Schmerzen bei lumbalem Bandscheibenvorfall eine intensive konservative Behandlung bis 12 Wochen empfehlen und frühe operative Verfahren lediglich bei Patienten mit beeinträchtigter Blasen‑/Mastdarmfunktion oder schwerer Kraftminderung empfehlen, gab es nach unserer Kenntnis bisher keine Studien, welche eine strukturierte kombinierte konservative Behandlung, wie in der vorliegenden Studie, untersucht haben. Die geringe Evidenz für die Wirksamkeit konservativer Behandlungsverfahren und die geringe Zahl durchgeführter Studien sind ein Grund für die Steigerung der Op.-Zahlen an der Wirbelsäule, welche nach Untersuchungen der Bertelsmann-Stiftung [30] von 2007 bis 2014 um 71 % von 450.000 auf 770.000 Eingriffe gestiegen sind, obwohl eine Überlegenheit gegenüber der konservativen Therapie nicht eindeutig nachgewiesen ist.

### Limitationen der Studie.

Da die Studie im klinischen Alltag eines Akutkrankenhauses durchgeführt wurde, war ein Vergleich zu einer unbehandelten Kontrollgruppe nicht möglich, was die Aussagekraft der Studie limitiert. Bei fehlender Verblindung und Randomisierung lässt sich auch ein Bias nicht ausschließen, obwohl mit der Zahl der im Beobachtungszeitraum erforderlichen Operationen, der Dauer der Arbeitsunfähigkeit und verschiedenen Fragebögen versucht wurde, eine untersucherbedingte Verfälschung der Ergebnisse zu minimieren. Unter Berücksichtigung der positiven Ergebnisse der vorliegenden Studie wäre die Durchführung einer größeren Studie mit Kontrollgruppe sinnvoll und bei Vergleichsstudien zwischen der konservativen und operativen Behandlung sollte ein kombiniertes konservatives Behandlungskonzept, wie hier dargestellt, eingesetzt werden.

## Fazit für die Praxis

Die konservative Kombinationstherapie mit mechanischer Physiotherapie (McKenzie), Gabapentin und transforaminalen epiduralen Infiltrationen ist beim lumbalen Bandscheibenvorfall mit Nervenwurzelreizsyndrom effektiv und wird gut toleriert.
